# Silencing FUT4 Inhibits the Progression of Osteosarcoma through Activation of FOXO1

**DOI:** 10.2174/0113816128269432240103052108

**Published:** 2024-02-27

**Authors:** Yang Yang, Xiaodi Yan, YueYuan Chen, Jiajia Liu, Jianhua Xue, Xiaoming Sheng, Jun Qin, Qiang Xue, Xianchen Liu

**Affiliations:** 1 Department of Trauma Center, Affiliated Hospital of Nantong University, Nantong City, Jiangsu Province 226001, China;; 2 Department of Radiation Oncology, Affiliated Hospital of Nantong University, Nantong City, Jiangsu Province 226001, China;; 3 Department of Oncology, Second People’s Hospital of Nantong & Affiliated Nantong Rehabilitation Hospital of Nantong University, Nantong City, Jiangsu Province 226001, China

**Keywords:** FOXO1, FUT4, osteosarcoma cells, proliferation, migration, apoptosis

## Abstract

**Background:**

It has been reported that inhibition of Fucosyltransferase4 (FUT4) to activate Forkhead box O1 (FOXO1) can lead to apoptosis of cancer cells, however, the mechanism in osteosarcoma is still unclear.

**Objective:**

To explore the biological significance of the connection between FUT4 and FOXO1 in osteosarcoma growth.

**Methods:**

* In vitro* tests were conducted using the human osteoblast cell line and the osteosarcoma cell lines. QRT-PCR assay as well as western blot assay were used to ascertain the relative expression levels of FUT4 and FOXO1 in the cells. By using the CCK-8 assay, colony assay, EDU assay, wound healing assay and Transwell assay, osteosarcoma cells' ability to proliferate, migrate and invade were examined in relation to si- FUT4. TUNEL test was used to evaluate Si-impact FUT4's on KHOS and U2OS apoptosis in osteosarcoma cells. Western blot assay was used to identify the expression of proliferative, migrating and apoptosis-related protein markers in osteosarcoma cells KHOS and U2OS and the expression of important proteins in the Wnt/ β-catenin signaling pathway.

**Results:**

In comparison with osteoblasts, osteosarcoma cells expressed more FUT4. The osteosarcoma cells' capacities to proliferate, invade, and migrate were markedly inhibited by the inhibition of FUT4 expression, which also increased osteosarcoma cell apoptosis. The Wnt/β-catenin signaling pathway was blocked by upregulating FOXO1 expression, which was in turn inhibited by inhibiting FUT4 expression.

**Conclusion:**

Osteosarcoma cells express more FUT4. The Wnt/β-catenin signaling pathway has a significant effect on osteosarcoma cell death, and inhibition of FUT4 expression may target FOXO1 activation to decrease osteosarcoma cells' ability to proliferate, invade, and migrate.

## 1 INTRODUCTION

Osteosarcoma, the most frequent primary malignant bone tumor in children and adolescents, is responsible for 8.9% of all cancer-related deaths in this age group. Over 50% of cases involve the knee and typically damage the long bone epiphysis [1]. Since 40% of clinical instances of osteosarcomas advance quickly and present with recurrence and/or metastasis with limited accessible therapy, it is recognized that all osteosarcomas are extremely malignant [2]. Currently, osteosarcoma is best treated with neoadjuvant chemotherapy, surgery, and high-dose chemotherapy. With an overall 5-year survival rate of little more than 30%, the prognosis for people with osteosarcoma remains dismal despite advances in diagnosis and therapy [3]. Finding new molecular targets is therefore crucial for developing efficient therapeutic approaches for the treatment of osteosarcoma [4].

A number of malignancies include the oncoprotein fucosyltransferase4 (FUT4) [5]. For instance, the Wnt/β-catenin pathway is regulated by the miR-29b/Sp1/FUT4 pathway, which controls acute myeloid leukemia (AML) [6]. MiR-125a-5p suppresses the development of bladder cancer in colorectal cancer *via* lowering the tumor suppressor FUT4 [7]. Through the NF-κB signaling pathway, MiR-26a and MiR-261b target FUT4 to influence the course of osteoarthritis [8]. LncRNAHOXB-AS1 controls AML through ELAVL1 and encourages the proliferation of multiple myeloma cells through the stability of FUT4 mRNA [9]. In addition, in a prior investigation, we discovered that human myeloma cells 143B cultured with modified exosomes loaded with miR-371b-5p had lower FUT4 levels than controls. However, the function and molecular processes of osteosarcoma, as well as the expression of FUT4 in osteosarcoma cells, remain unclear and still require additional research.

FOXO1, FOXO2, FOXO3, and FOXO4 are members of the FOX family [10]. As a member of the FOXO family and a crucial transcription factor, FOXO1 was discovered to be one of the most significant AKT substrates [11]. According to one study, activated AKT may phosphorylate FOXO1 at three separate serine/threonine sites, which prevents FOXO1 from moving into the nucleus. Numerous target genes involved in apoptosis, autophagy, and cell cycle arrest are modulated by the AKT/FOXO1 signaling pathway, which controls many biological processes [12]. It has been demonstrated that activating FOXO1 by suppressing FUT4 can cause apoptosis in non-small cell lung cancer [13]. Nevertheless, the mechanism behind how FUT4 and FOXO1 interact in osteosarcoma is unknown and requires more research. Investigating the biological role and probable mechanism of the connection between FUT4 and FOXO1 in the development of osteosarcoma was the aim of this investigation.

## 2 MATERIALS AND METHODS

### 2.1 Cell Culture

In addition to human osteoblasts (hFOB1.19), the American Type Culture Collection (ATCC) (Ma, USA) also supplied human osteosarcoma cell lines (SaOS2, 143B, KHOS, and U2OS). All of the cells were cultured in Dulbecco's modified Eagle medium (Sigma) with 10% fetal bovine serum (FBS) (Ausbian), 0.1 mg/mL streptomycin, and 100 U/mL penicillin at 37°C in a 5% CO_2_ incubator.

### 2.2 Cell Transfection

About 2×10^5^ hFOB1.19, SaOS2, 143B, KHOS, and U2OS cells were inoculated and grown in 6-well plates prior to transfection. Cells were transfected with reference to the Lipofectamine 3000 instructions (L3000015; Thermo Fisher Scientific, Inc.). They were divided into blank transfection group named si-NC and transfection group named si-FUT4.

### 2.3 RNA Extraction

The NucleoSpin RNA kit (Macherey-Nagel, UK) was conducted to extract RNA from cells (5 × 10^6^ cells) in accordance with a small RNA purification methodology. The Agilent 2100 Bioanalyzer, which is well suited for short RNA (length < 200 nucleotides), was adopted to analyze extracted RNA samples using Agilent 2100 Expert software. By performing a smear analysis on the electropherograms produced by capillary electrophoresis, RNA integrity and quantity were evaluated.

### 2.4 qRT-PCR Assay

Following the manufacturer's guidance and instructions, QRT-PCR analysis of miRNA expression was carried out using the TaqMan MicroRNA Assay Kit (Applied Biosystems, USA). Using the TRIzol reagent, total RNA was quickly extracted from tissues and cells (Invitrogen, USA). Then, using gene-specific primers, total RNA was converted into cDNA. The 10 ng RNA sample, 50 nmol/L stem-loop RT primer, 1x RT buffer, 0.25 mmol/L of each dNTP, 3.33 U/μL MultiScribe reverse transcriptase, and 0.25 U/μL RNase inhibitor were the components of the reverse transcriptase reactions. 15 μL of the reactions were incubated for 1 hour at 16°C, followed by 30 minutes at 42°C, 30 minutes at 85°C, and 5 minutes at 4°C. 20 μL of PCR reactions contained 1.33 μL of RT product, 1× of TaqMan Universal PCR master mix, and 1 μL of the primer and probe mixture from the TaqMan MicroRNA Assay kit. For 40 cycles of 15 s at 95°C and 1 min at 60°C, reactions were incubated on 96-well optical plates for 10 min at 95°C. On an Applied Biosystems 7500 real-time PCR equipment, PCR reactions were carried out, and 7500 System SDS software was used to evaluate the results. In Table **1**, the list of primer sequences is displayed.

### 2.5 Western Blot Assay

RIPA lysate (R0278; Sigma-Aldrich; Merck KGaA) and a protease inhibitor were used to lyse cells (1×10^6^) and extract total protein (S8830; Sigma-Aldrich; Merck KGaA). Each sample's total protein (50 µg per lane) was separated on a 10% SDS-PAGE at 120 V for around 1.5 hours. The protein suspension was mixed with 1X antibody solution, which was then transferred to a PVDF membrane and sealed with 5% skim milk for 1 hour at room temperature. The cell membranes were sealed with skim milk and then treated with a primary antibody overnight at 4°C (Sigma-Aldrich; Merck KGaA). The membranes were incubated with the matching secondary antibodies for 1 hour at room temperature the next day, after being washed with Tris-buffered saline and Tween 20 (TBST; Sigma-Aldrich, St. Louis, MO, USA). To find the proteins, Cell Signaling Technology, Inc.'s SignalFire™ ECL reagent (Catalog #6883) was used. Protein blot results were optical density analyzed and quantified using ImageLabTM software (version 3.0) from Bio-Rad Laboratories Inc.

### 2.6 cck-8 Assay

First, 1 × 10^4^ cells/well of 96-well plates were injected with cells. Following cell adhesion, each well received 100 μL of media with 10 μL of CCK-8 (Beyotime, Shanghai, China). Then, a recording of the optical density (OD) was made at 450 nm after 1 hour.

### 2.7 Colony Formation Assay

A 6-well plate of single cells was used to cultivate the collected, 0.25% trypsin-digested, and harvested cells in the log phase. When a clear clone is seen in the culture plate, the culture is stopped. Cells were gently washed twice with PBS after the supernatant was discarded. The cells were then fixed using 5 mL of 4% paraformaldehyde for 15 min. The dye solution is then added to the stain for 10 to 30 minutes after the fixation solution has been withdrawn and the proper quantity of GIMSA has been applied. The dye solution was removed with running water, and the area was then air-dried. The grid holding the transparent film is then overlaid on the plate after it has been inverted. Macroscopically count the number of clones, or count clones with at least 10 cells using a low-power microscope. Lastly, figure out how quickly clones form.

### 2.8 EdU Assay

Using the cell Light EdU DNA Cell Proliferation Kit (RiboBio, Guangzhou, China), EdU assay was conducted to determine the proliferation of cells. Transfected cells were exposed to 50 mM EdU and they were incubated for an additional 2 h after the incubation for 48 h at 37°C and 5% CO_2_. Next, stain the proliferating cells with an Apollo dye solution after the cells had been fixed with 4% paraformaldehyde. All cells’ nucleic acids were stained with DAPI. By the program ImageJ (Version 1.8.0; National Institutes of Health, Sacaton, AZ, USA), cell proliferation rates were estimated. By a fluorescent microscope, pictures were captured.

### 2.9 Wound Healing Assay

To assess cell motility, wound-healing assay was performed. Cells were inoculated on 6-well plates and cultured to 100% confluence. A 200 μL pipette tip was used to scratch the injured monolayer, which was subsequently rinsed with PBS and cultured in serum-free media. Using an inverted microscope, the rate of wound healing was assessed after 48 hours (BX53, Olympus). Using ImageJ, images were examined and quantified (NIH, USA).

### 2.10 Transwell Assay

The invasive potential of the cells was investigated using a transwell system (Corning, USA) with a 0.4 μm chamber. Using an Olympus microscope to capture images of the invasive cells, the number of cells in 5 randomly selected areas on the lower membrane surface was calculated.

### 2.11 TUNEL Assay

Cells were raised twice in PBS before being fixed and kept for 15 minutes in 4% paraformaldehyde. Then, they are permeabilized for 20 minutes in 0.25% Triton-X 100. The TUNEL assay was carried out with reference to the manufacturer’s recommendations and instructions (Roche). In short, cells were treated with a click reaction mixture after being initially incubated at 37°C for 45 min in a reaction mixture that contained terminal deoxynucleotidyl transferase (TdT). Hematoxylin or methyl green was adopted to stain the cell nuclei.

### 2.12 Statistical Analysis

The Statistical Products and Services Solutions (SPSS) 20.0 software (IBM, Armonk, NY, USA) was used to conduct all data analyses. All experiments were performed in triplicate in parallel, and the results of the assay were given as mean ± standard deviation. Group differences were examined using the t-test. One-way and multi-way ANOVAs were used to assess differences between different groups. At *p <* 0.05, it was statistically significant.

## 3 RESULTS

### 3.1 Elevated Expression of FUT4 in Human Osteosarcoma Cells

**Fig. (1):**
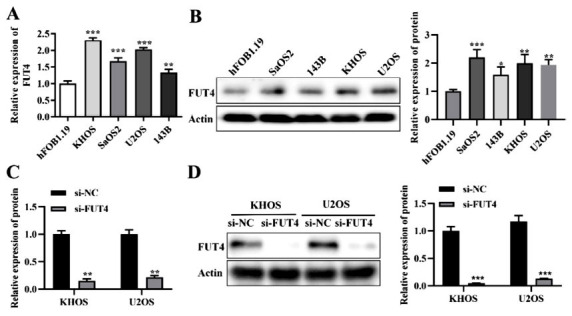
Human osteosarcoma cells express FUT4. (**A**) FUT4 expression levels in hFOB1.19, KHOS, SaOS2, U2OS, and 143B were measured at the RNA level. (**B**) FUT4 expression levels in hFOB1.19, KHOS, SaOS2, U2OS, and 143B were measured at the protein level. (**C**) FUT4 expression levels in KHOS and U2OS cells following FUT4 transfection were measured at the RNA level in KHOS, U2OS. (**D**) FUT4 expression levels in KHOS and U2OS cells following si-FUT4 transfection were measured at the protein level. *p < 0.05, **p < 0.01, ***p < 0.001.

Findings from qRT-PCR assay and western blot assay displayed that human osteosarcoma cells KHOS, SaOS2, U2OS, and 143B expressed more FUT4 than the osteoblast cell line (hFOB1.19) (Figs. **1A** and **1B**). In comparison to SaOS2 and 143B, FUT4 expression was comparatively greater in KHOS and U2OS. KHOS and U2OS cell lines were therefore chosen for the research that came next. KHOS and U2OS cells were transfected with si- FUT4. Results from qRT-PCR assay and western blot assay exhibited that si-FUT4 transfection dramatically reduced the expression of FUT4 in KHOS and U2OS cells (Figs. **1C** and **1D**).

### 3.2 Inhibition of FUT4 Expression Prevents Osteosarcoma Cells from Proliferating, Invading and Migrating

**Fig. (2):**
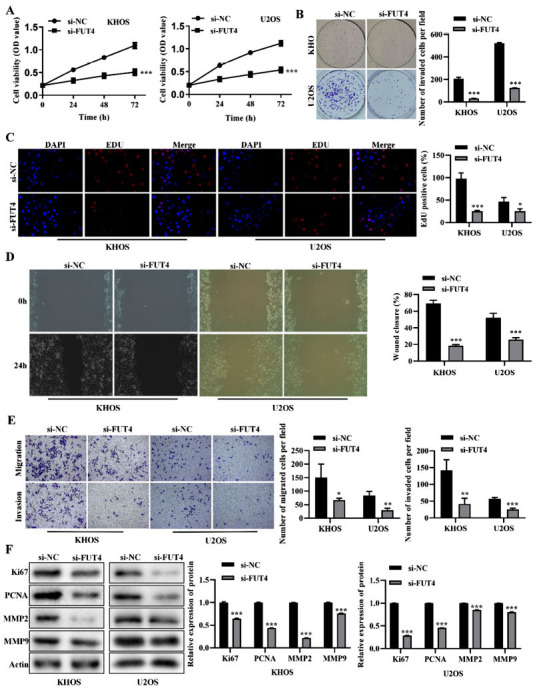
Effect of si-FUT4 on human osteosarcoma cell’s progressions of proliferating, invading, and migrating. (**A**) Effect of si-FUT4 on KHOS and U2OS cell viability. (**B**) Effect of si-FUT4 on colony number in KHOS and U2OS cells, with typical photos and quantitative analysis findings shown on the left and right, respectively. (**C**) Effect of si-FUT4 on KHOS and U2OS cell proliferation (EdU). On the right, you can see representative fluorescence pictures and the findings of a quantitative analysis. (**D**) Analysis of the impact of si-FUT4 on KHOS and U2OS cell migration using a wound healing test. The findings of the quantitative analysis are shown on the right, while representative photographs are shown on the left. (**E**) Analysis of the Transwell assay's findings regarding si-impact FUT4's on KHOS and U2OS cell invasion and migration. The representative images are on the left, while the quantitative analysis findings are on the right. (**F**) Effect of si-FUT4 on the expression of Ki67, PCNA, MMP2, and MMP9. The protein utilized as a control was actin. The findings of a relative quantitative analysis are shown on the right side, while the left side displays illustrative images. *p < 0.05, **p < 0.01, ***p < 0.001.

Results from CCK-8 demonstrated that si-FUT4 drastically reduced the viability of human osteosarcoma cells (Fig. **2A**). Si- FUT4 dramatically decreased the growth of human osteosarcoma cells, according to a colony formation experiment (Fig. **2B**). Further EdU analysis revealed that si-FUT4 transfected human osteosarcoma cells had a substantial reduction in proliferation (Fig. **2C**). According to the results of a wound healing experiment, si- FUT4 dramatically reduced the migration of human osteosarcoma cells (Fig. **2D**). Si-FUT4 considerably reduced the invasion and migration of human osteosarcoma cells, according to the results of the transwell experiment (Fig. **2E**). Proliferation-related proteins Ki67 and PCNA, as well as migration-related proteins MMP2 and MMP9, were all dramatically downregulated by si-FUT4 (Fig. **2F**).

### 3.3 Inhibition of FUT4 Expression can Promote Apoptosis in Human Osteosarcoma Cells

**Fig. (3):**
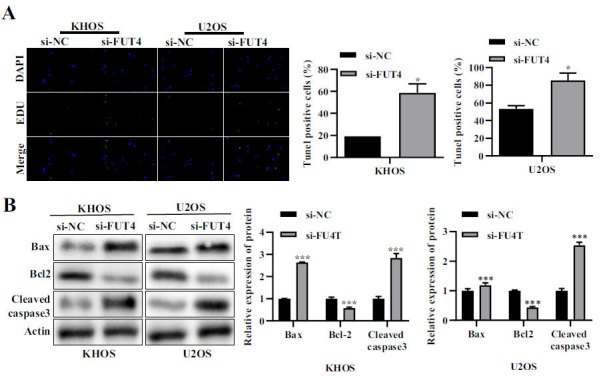
Effect of si-FUT4 on human osteosarcoma cell apoptosis. (**A**) Effect of si-FUT4 on KHOS and U2OS cell apoptosis. The representative fluorescence pictures are shown on the left, while the quantitative analysis findings are shown on the right. (**B**) Effect of si-FUT4 on Bax, Bcl2 and Cleaved caspase3 Expressions. The protein utilized as a control was actin. On the left are the representative images, and on the right are the findings of the relative quantitative analysis. *p < 0.05, **p < 0.01, ***p < 0.001.

Inhibiting FUT4 expression greatly accelerated apoptosis in human osteosarcoma cells, according to the TUNEL assay's findings (Fig. **3A**). The pro-apoptotic proteins Bax and Cleaved caspase3 were dramatically upregulated in KHOS and U2OS cells when FUT4 expression was inhibited, whereas the inhibitory protein Bcl-2 was significantly downregulated (Fig. **3B**).

### 3.4 FUT4 Regulates the Expression of FOXO1 Gene

**Fig. (4):**
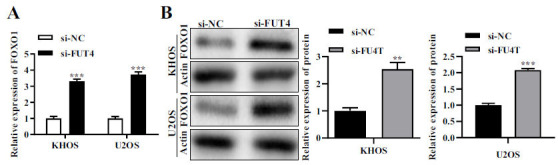
The impact si-FUT4's on FOXO1 gene expression. (**A**) Effect of si-FUT4 on the RNA level of FOXO1 expression in KHOS and U2OS cells. (**B**) Effect of si-FUT4 on FOXO1 expression levels in KHOS as well as U2OS cells at the protein level. The protein utilized as a control was actin. The findings of a relative quantitative analysis are shown on the right, while representative photographs are shown on the left. *p < 0.05, **p < 0.01, ***p < 0.001.

Findings from qRT-PCR assay and western blot assay exhibited that si-FUT4 finely transfected KHOS and U2OS had considerably higher FOXO1 expression (Figs. **4A** and **4B**).

### 3.5 FUT4/FOXO1 can Regulate the Expression of Key Molecules in Wnt/β-catenin Signaling Pathway

**Fig. (5):**
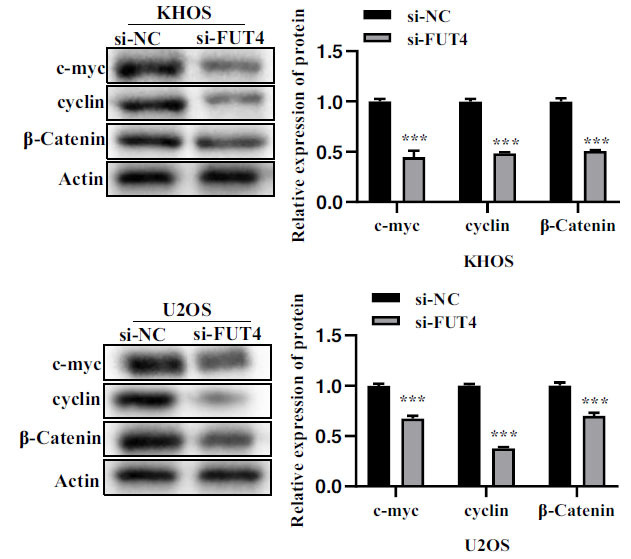
si-effects FUT4's on the Wnt/β-catenin signaling pathway. Effect of si-FUT4 on the protein level of c-myc, cyclin, and β-catenin expression in KHOS and U2OS cells. The protein used as a control was actin. Results of a relative quantitative analysis are shown on the right, and representative images are exhibited on the left. *p < 0.05, **p < 0.01, ***p < 0.001.

Western blot assay exhibited that the expression of c-myc, cyclin and β-catenin in the Wnt/β-catenin signaling pathway was significantly reduced in KHOS and U2OS cells transfected with si- FUT4 (Fig. **5**).

## 4 DISCUSSION

Osteosarcoma is significantly less prevalent than many other malignancies, yet it is a malignant tumor [14]. Adolescents are more likely to develop osteosarcoma in the proximal tibia or distal femur [15]. Regarding the precise etiology of osteosarcoma, there are still many unsolved questions. Numerous research on molecular pathways have been conducted recently, and they have helped to understand how osteosarcoma develops [16]. Numerous genes, miRNAs, and lncRNAs have been linked to the progression of osteosarcomas due to advances in genomics [17, 18]. Fucosyltransferase 4 (FUT4) is known to play a role in osteosarcoma cell proliferation and metastasis [18].

We first assessed the levels of FUT4 expression in osteoblasts and osteosarcoma cells in this study. The results showed that osteosarcoma cells expressed more FUT4 than osteoblasts, potentially implicating FUT4 in the emergence of osteosarcoma. Due to their relatively higher levels of FUT4 expression, we selected KHOS and U2OS cells as *in vitro* experimental models to further clarify the biological roles of FUT4. According to earlier studies, FUT4 may prevent cisplatin resistance in lung cancer from being caused by FOXO1-induced apoptosis [13]. The findings of the current study demonstrated that inhibiting FUT4 expression significantly reduced the abilities to proliferate, invade and migrate osteosarcoma cells while favoring osteosarcoma cell death. FUT4 silencing can lead to apoptosis and inhibit the proliferation of A549 and H1975 cells by blocking the phosphorylation of Akt and FOXO1 and nuclear translocation of FoxO2 induced by cisplatin treatment. We hypothesize that there could be some interaction between FUT4 and FOXO1, which is crucial for osteosarcoma growth and metastasis. According to the current study's findings, FOXO1 expression can be increased by inhibiting the expression of FUT4. Previous research has demonstrated that abnormal Wnt signaling is linked to human osteosarcoma, suggesting a genetic vulnerability that may be exploited in osteosarcoma therapy [19]. Inhibition of FUT4 expression in this study restrained the Wnt/β-catenin signaling pathway and hence the osteosarcoma cells' capacities to proliferate, invade and migrate, which is consistent with earlier results [20].

This research suggests that FUT4 regulates Wnt/β-catenin signaling to affect osteosarcoma cells. Wnt/β-catenin signaling is a major androgen signal, and the androgen receptor has also been reported in osteosarcoma, is considered as a novel potential prognostic marker in osteosarcoma [21] and has interaction with another important steroid receptor, FXR [22], which in turn is an important regulator of FOXO and tumorigenesis [23]. Similarly, estrogen is also a FUT4 modulator [24] and has been described to affect osteosarcoma growth [25]. The potential role of sex hormones in osteosarcoma needs further investigation.

## CONCLUSION

FUT4 plays a crucial role in the progression of osteosarcoma. Osteosarcoma cells are prevented from proliferating, invading, or migrating when FUT4 expression is inhibited. This action also activates FOXO1 expression and significantly inhibits the Wnt/β- catenin signaling pathway, which offers a potential therapeutic target for the treatment of osteosarcoma in humans.

## AUTHORS’ CONTRIBUTIONS

XCL conceived and designed the study. YY, XDY and YYC conducted most of the experiments. JHX, XMS and JJL analyzed the data. JJL performed the literature search and data extraction. XCL and JQ drafted the manuscript. XCL and QX finalized the manuscript. All authors read and approved the final manuscript.

## Figures and Tables

**Table 1:** Primer sequences.

**Gene**	**Forward (5′-3′)**	**Reverse (5′-3′)**
FOXO1	TGGACATGCTCAGCAGACATC	TTGGGTCAGGCGGTTCA
FUT4	AAGGTCCAGGCCCACTGAAG	CAGTTCAGGTGACAGAGGCTCA
GAPDH	ATGGGGAAGGTGAAGGTCG	GGGGTCATTGATGGCAACAATA

## Data Availability

All data generated or analyzed during this study are included in this published article and are available from the corresponding author (XL), upon reasonable request.

## LIST OF ABBREVIATIONS

AML: Acute Myeloid Leukemia
FBS: Fetal Bovine Serum
FUT4: Fucosyltransferase4
OD: Optical Density
TdT: Terminal Deoxynucleotidyl Transferase
